# Low frequency piezoresonance defined dynamic control of terahertz wave propagation

**DOI:** 10.1038/srep38041

**Published:** 2016-11-30

**Authors:** Moumita Dutta, Soutik Betal, Xomalin G. Peralta, Amar S. Bhalla, Ruyan Guo

**Affiliations:** 1 Department of Electrical and Computer Engineering, University of Texas at San Antonio, San Antonio, TX 78249, USA; 2 Department of Physics & Astronomy, University of Texas at San Antonio, San Antonio, TX 78249, USA

## Abstract

Phase modulators are one of the key components of many applications in electromagnetic and opto-electric wave propagations. Phase-shifters play an integral role in communications, imaging and in coherent material excitations. In order to realize the terahertz (THz) electromagnetic spectrum as a fully-functional bandwidth, the development of a family of efficient THz phase modulators is needed. Although there have been quite a few attempts to implement THz phase modulators based on quantum-well structures, liquid crystals, or meta-materials, significantly improved sensitivity and dynamic control for phase modulation, as we believe can be enabled by piezoelectric-resonance devices, is yet to be investigated. In this article we provide an experimental demonstration of phase modulation of THz beam by operating a ferroelectric single crystal LiNbO_3_ film device at the piezo-resonance. The piezo-resonance, excited by an external a.c. electric field, develops a coupling between electromagnetic and lattice-wave and this coupling governs the wave propagation of the incident THz beam by modulating its phase transfer function. We report the understanding developed in this work can facilitate the design and fabrication of a family of resonance-defined highly sensitive and extremely low energy sub-millimeter wave sensors and modulators.

In the last few decades we have witnessed a substantial development in THz technology. With the advancement in femtosecond laser technology and the coherent generation and detection of THz radiation^1^,^2^,^3^,^4^,^5^,^6^,^7^,^8^,^9^,^10^,^11^,^12^,^13^, the previously unexplored gap (ranging from 0.1 THz to 30 THz) is now finding applications in various disciplines^14^,^15^. This spectral region has now become accessible to numerous applications and fundamental studies such as imaging, communications, explosive detection, spectroscopy of molecular vibrations, and precision control of spin and lattice waves^16^,^17^,^18^,^19^,^20^,^21^, to name a few.

However, in order to incorporate terahertz wave devices into full-fledged applications, efforts need to be directed towards advancing the design and implementation of efficient THz modulators and switches in order to fully control and manipulate the THz wave. Quite a few attempts, as was well summarized in ref. 22, have been made to come up with efficient architectures. Different modulation techniques have been tested for that matter, all-optical^23^, electronic^24^, and thermal^25^ being the significant ones, along with the utilization of some promising candidate materials such as graphene^26^ and photonic crystals^27^.

Phase shifters or modulators that can be tuned dynamically are of fundamental importance for numerous devices and applications in telecommunication and wave processing systems such as phased array antennas, isolators, filters, absorbers, frequency shifters, spectral lensing, amplifiers, lasers and so on. Owing to the accompanying limitations of other techniques, such as the need for cryogenic temperatures of operation^28^, low speed^29^, most attempts have been focused on using metamaterials. The first experimental demonstration of a room-temperature solid state metamaterial THz phase modulator was reported by Chen and group in 2009^30^. However, there has been no concerted effort to exploit monolithic yet tunable materials as the basis for THz phase modulators.

In this research, we demonstrate how a single crystalline ferroelectric material, LiNbO_3_, operated at the piezoelectric resonance condition, can be designed to induce a strong lattice-phonon: polarization-gradient coupling effect that defines the propagation of the incident terahertz waveform by modulating its phase. This approach presents multiple advantages over alternate methods. In contrast to metamaterials and quantum-well structures, it does not involve any complex assembly or fabrication process. In addition, it does not depend on specific temperature or phase transitions for its functionality and, most of all, it provides dynamic scalability as far as the frequency of operation and the required phase modulation are concerned.

The indices of refraction of a material are the most effective parameter that governs its interaction with electromagnetic (EM) waves and can be tailored for shaping and modulating them. For example, engineered periodic ferroelectric domains have been widely used for quasi-phase matching in devices for second harmonic generation^31^. In materials exhibiting anisotropic symmetry and operating at the piezoelectric resonance, the gradient of polarization vectors 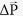 oscillates in such a manner that the equivalent circuit of the material encounters an impedance that varies from minimum to maximum at the resonant and anti-resonant nodes respectively. Therefore, the localized refractive index undergoes jumps from a maximum to a minimum, incorporating a red and a blue shift in the dispersion of the interacting electromagnetic (EM) wave, which can be expressed in the form of a phase change. Although experimental evidence and empirical understanding of this phenomenon exist to substantiate the perception that such resonant interactions can dramatically affect EM wave propagation^32^,^33^,^34^, due to the complexity of characterizing and modelling materials at resonance, there is limited analytical explanations describing the sub-millimeter EM wave-matter interactive behavior under such singularity conditions. In this article we provide, for the first time to our knowledge, an experimental demonstration of dynamic control of terahertz wave propagation via the piezoelectric resonance of a single crystal ferroelectric thin film. The phase transfer function is modulated by the spatial variation of the refractive index induced by the piezoelectric resonance in a multi-layered single crystal thin film system, which is excited by an applied low frequency electric field.

## Results

### Structure and surface morphology analysis

**Figure 1:**
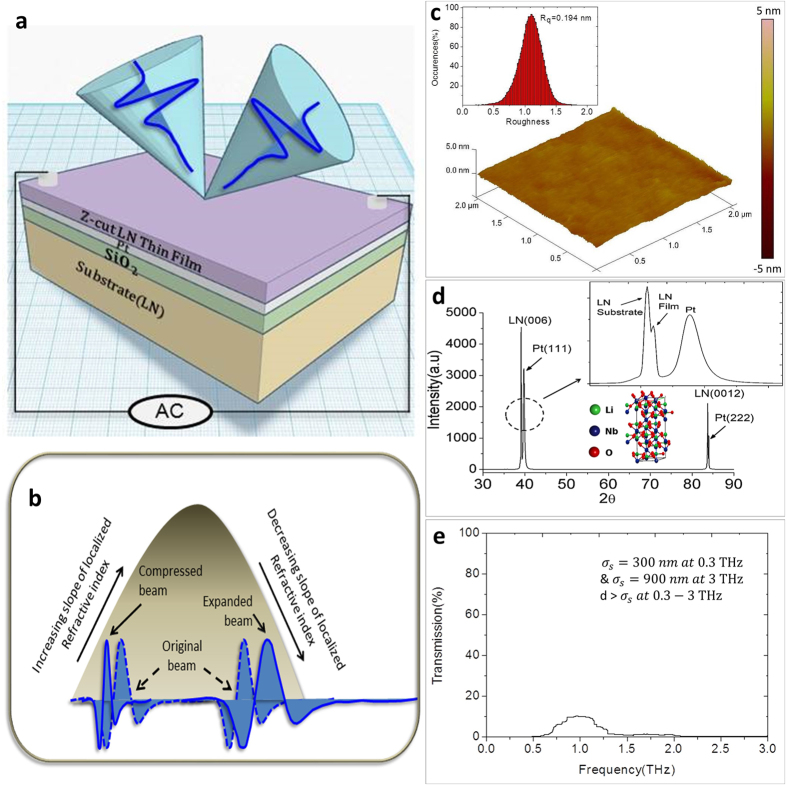
Experimental-setup, the crystallographic orientation and morphology of the multilayered single crystal LN thin film system employed for THz wave modulation. (**a**) Schematic of the multilayered thin film system comprising an ion-sliced single crystal z-cut LN (504 nm thick) deposited on platinized silica grown on an LN substrate (500 μm thick). The top layer is excited by applying an external a.c. field via two silver electrodes deposited at opposite corners diagonally placed on the surface. (**b**) Illustration of the reshaping of a THz wave due to its interaction with a piezoelectric crystal whose index of refraction is subject to a localized polarization gradient. The wave gets compressed at the positive slope of the index and expanded at the negative slope of the index. (**c**) AFM micrograph (2 μm × 2 μm) of the surface of the thin film system. It depicts a smooth surface topography with the roughness having a root mean square value of (Rq) ~ 0.194 nm as shown in the inset. (**d**) XRD spectrum confirms the crystal orientation of the LN thin film system, deposited on Pt (111), being Z-axis (00 *l*) in pure rhombohedral phase (with the simulated crystal structure in the inset). Pure phase of both the substrate and the top layer of LN are prominently visible in the peak doublets (see the inset). (**e**) THz transmission spectra of the platinized thin film system. As calculated from the skin depth *σ*_*s*_ of platinum,the embedded 400 nm thick layer is opaque to THz waves resulting in a very low transmission amplitude therefore making the system suitable for reflection mode of operation where the loss of incident energy due to transmission is low.

The thin film system used for the phase-modulator design presented in this work (as depicted in Fig. 1), comprises a single-crystal Z-cut LiNbO_3_ (LN) having a dimension of 1.2 cm × 1.3 cm. As described in ref. 35, the top LN layer (of 504 nm) was peeled off from its parent single crystal by using ionic implantation and wafer bonding technique, prior to being implanted on a thin platinum layer (400 nm) using magnetron sputtering. The platinized LN is then bonded onto the parent LN substrate (500 μm) with a plasma-enhanced chemical vapor deposited SiO_2_ (1700 nm) layer in between.

The surface morphology was verified by using atomic force microscopy (AFM) topographical imaging (using Veeco Multimode V), configured at tapping mode. As observed from the AFM micrograph (shown in Fig. 1(c)), the surface bears a smooth morphology with a root-mean-square roughness (Rq) of ~0.194 nm which essentially makes any contribution onto the phase modulation due to surface topology insignificant.

The X-ray-diffraction (XRD) pattern was obtained to reveal the film device’s crystallographic-phase and orientation, along with its chemical composition. The strong (00*l*) diffraction peaks, observed in the XRD measurement of the thin film (see Fig. 1(d)), confirm the rhombohedral phase of LN, while the peak doublets, e.g., the (00*6*) line, are attributed to the substrate and the film respectively. The crystallinity of the embedded metallic platinum (Pt) layer is also shown to be epitaxially grown on SiO_2_ with (111) orientation. The high crystalline quality of the film (with no sign of phase impurity or domain walls) has been verified, confirming it to be free from any crystallographic and morphological discontinuities, which ensures its suitability for application as a high density device.

### Phase Modulation of the coupled THz wave

The phase modulation of the incident THz wave 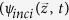, having a spectral range *ω*_*THz*_/2π → 0.1–3 THz) was realized by applying a low frequency electric field to the LN thin film as the external excitation, while the operational temperature was maintained at 300 K. As illustrated in Fig. 1(a) and more elaborately described in the Methods section, the top layer of the thin film system was subjected to an a.c. E-field excitation with a peak-to-peak amplitude of 10 V_p-p_ and a set of frequencies ranging from 19.2 kHz to 20.9 kHz (*E*_*ac*_|_*ω*→19.2–20.9*kHz*_). *E*_*ac*_ was applied across the surface of the thin film via two silver electrodes deposited at two diagonally opposite corners. The electrodes were so positioned to avoid any direct obstruction to the interaction between the surface of the thin film and the THz wave. The THz was normally incident onto the surface of the film. In order to isolate the genuine influence of piezoresonance and avoid any edge effects, the entire THz beam (Full width at half maximum (FWHM) ~1 mm) was localized within the central region of the film. While the thin film was excited by an external a.c. field of a particular frequency, the resulting reflected THz time domain response 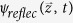 was recorded using a THz time domain spectrometer (THz TDS), where the incident THz beam corresponded to a spectral range of 0.1 to 3 THz. The THz measurement was repeated each time after changing the E_ac_ frequency by steps of 0.1 kHz.

It should be noted that before using the THz-TDS in reflection mode, the THz transparency of the sample was examined by performing transmission mode spectroscopy. As shown in Fig. 1(e) the thin film system exhibits a THz transmission 
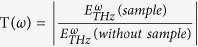
 of only ~10%. Platinum exhibits a skin depth σ_s_ of 300 nm at 0.3 THz and 90 nm at 3 THz; therefore, given the fact that the embedded platinum layer is 400 nm thick, it is opaque to THz waves rendering the system qualified for reflection mode spectroscopy. Detailed THz characterization is given in Supplemental Information S1.

The responses corresponding to each of the excitation frequencies 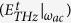 captured by the reflected terahertz pulses 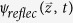 were then examined by an extensive analysis for the induced effect. To correlate the changes observed as a function of excitation frequency, the temporal components of the reflected THz wave were converted into the frequency domain 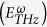 by a Fourier transformation in order to obtain their spectral phase 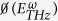 and amplitude 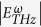 information.

**Figure 2:**
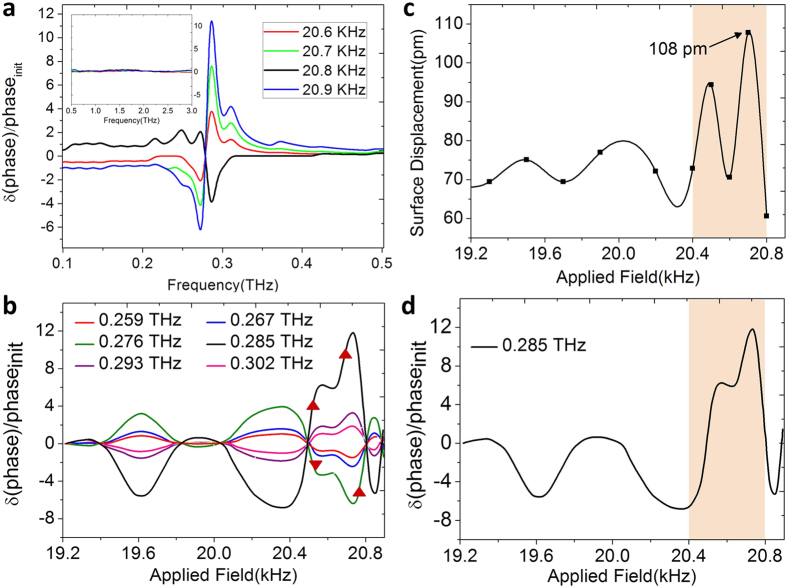
Evidence of resonance induced phase modulation. (**a**) Applied field dependent relative phase response of the reflected terahertz wave, normalized with respect to the response observed without applying any external field. Phase response starts from 20.6 kHz with a phase reversal occurring at 20.8 kHz. Prominent field-dependent excitations are observed only in the frequency range of 0.1–0.5 THz, beyond which no significant response is observed (see inset). (**b**) Relative THz phase response plotted against the frequency of the applied external field. The relative phase response appears to exhibit a rippling surface-wave like behavior. The phase-shift is apparent at 0.259 THz and increases gradually until 0.276 THz. A sudden phase reversal relative to that at 0.276 THz was observed at 0.285 THz along with an indication of maximum response. For higher THz frequencies, the relative phase response flattens out and disappears completely beyond 0.302 THz. (**c**) Contour of the surface displacement profile measured with a vibrometer for excitation frequencies ranging between 19.2 kHz to 20.9 kHz. The surface displacement profile shows the maximum displacement of 108 pm at 20.7 kHz indicating it to be the regime of piezo-response along with rendering correspondence to (**d**), the THz phase response observed at 0.285 THz.

For each of the E-field frequencies applied, *E*_*ac*_|_*ω*_, the field-induced relative phase responses, 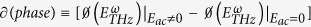, were calculated from the spectral components of the reflected terahertz beam. The relative phase response ∂(*phase*) was normalized with respect to the response observed without applying any external field 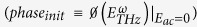. As observed in Fig. 2(a), the *E*_*ac*_ frequency dependent phase response 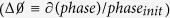 started prominently from 20.6 kHz which increases in amplitude as the *E*_*ac*_ frequency increases, but undergoes a phase reversal (phase-shift of π) at 20.8 kHz 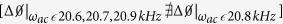 (where 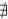 signifies anti-parallelism and 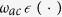 denotes at a particular applied excitation frequency). Note that the excitations were observed only in the range of 0.1–0.5 THz above which the phase responses appear to get flattened out (see the inset of Fig. 2(a)).

As a next step, the frequency window (0.1–0.5 THz) in which the prominent phase responses were observed was examined in more detail. The relative phase shifts at a given THz frequency 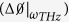 were plotted as a function of the frequency of the applied excitation field *E*_*ac*_|_*ω*_. As shown in Fig. 2(b), the response starts cropping up from 0.259 THz and increase in magnitude until 0.276 THz. At 0.285 THz the phase reverses, and the response is maximal. For frequencies above 0.285 THz, the response tapers down and vanishes mostly beyond 0.302 THz 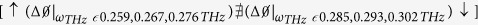 (where ↑ and ↓ signifies gradual increase and decrease of response respectively). Moreover, the phase response seems to exhibit a rippling surface-wave like behavior as prominently exhibited in Fig. 2(b).

### Piezoelectric resonance and its induced effect

In order to further investigate the dependence of the applied field to the dynamic phase response observed, the piezoresonance frequency of the system was measured. For a quantitative assessment of its electromechanical coupling effect, a vibrometric surface displacement evaluation technique was employed. An a.c. electric field similar to that used for terahertz phase manipulation, was applied via the two surface electrodes (the experimental details are elaborated further in the Methods section). The excitation field of a given freqnency ω was applied in-plane (*E*_*ac*_|_*ω*_. ⊥ c-axis) while the out of plane surface displacement 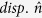 (where 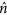 //c-axis) was measured as a function of the excitation frequencies applied. From the surface displacements measured, a maximum deflection of 108 *p*m was found to occur at 20.7 kHz corresponding to its characteristic regime of piezoelectric resonance. The displacement of 108 *p*m for the top LiNbO_3_ layer (504 nm thick) yields an out-of-plane strain of 214 parts per million. Moreover, the profile of the surface displacement (as shown in Fig. 2(c)) has a clear correspondence to the phase modulation contour observed at 0.285 THz (refer to Fig. 2(d)).

To interpret and corroborate the experimental outcomes, finite element analysis (FEA) simulations have been performed using conditions prescribed in these experiments. Commercially available COMSOL Multiphysics suite 4.4 (COMSOL Inc.) was employed for this purpose. A frequency domain analysis was performed using the physics of piezoelectric devices, where a scan over a spectral range of 19 kHz to 25 kHz was executed.

**Figure 3:**
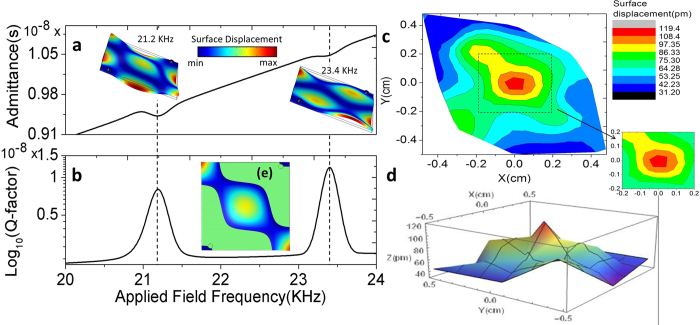
Vibrometry results and finite element simulation corroborating the experimental observation. Finite element simulations over a spectral window of 19 kHz to 25 kHz (performed by implementing prescribed experimental conditions) show two resonances occurring at 21.2 kHz and at 23.4 kHz which are reflected in (**a**) the admittance and (**b**) quality factor plots. The insets are 3D representations of the surface displacements simulated at the two resonant conditions. (**c**) 2D contour plot and (**d**) 3D surface plot of a set of 25 vibrometer data points measured at the resonant frequency of 20.8 kHz, with the marked region representing the central part of the film where the THz beam is focused on. (**e**) Simulated surface-contour displacement plot at 21.2 kHz. The correspondence with (**c**) confirms that the corners where the surface displacement is independent of the excitation are the regions hosting the electrodes.

As shown in Fig. 3, the FEA simulations are in very good agreement with the experimental results. The FEA simulated admittance and quality factor show a resonant behavior at two frequencies with the primary piezoresonance occurring at 21.2 kHz and the secondary one at 23.4 kHz (see Fig. 3(a) and (b)). The frequency of the simulated primary resonance lays within ~2% of that observed experimentally (i.e., at 20.8 kHz where the THz phase response showed a phase reversal as depicted in Fig. 2 (a)), providing credible support towards its physical origin. The vibrometer surface profilometric measurement (see Fig. 3(c) and (d)) shows that the maximum displacement occurs at the center of the sample and spreads out along two diagonal directions. Near the other two corners where the electrodes were positioned, the displacement was minimal. The vibrometer surface profilometric measurement was obtained by probing 25 points across the surface of the sample while keeping the excitation frequency at 20.8 kHz. A correspondence is also observed between the 2D contour plot of the vibrometer surface displacement profilometric measurement and the surface-contour displacement plot at 21.2 kHz as obtained from the FEA simulations, see Fig. 3(c) and (e) respectively. The deviation observed between the experimental and simulated results, however small it may be, can be attributed to the inherent limitations in reproducing the exact experimental boundary conditions in the simulations. As can be observed from the 2D contour plot (see the marked region of Fig. 3(c)), the surface displacements brought about by the piezoelectric response at the center of the sample, is sufficiently effective to introduce perturbation in the propagation of the interacting THz beam (FWHM ~1 mm) and to tailor its phase. It is the excitation-field induced surface-strain leading to differential contrast in dielectric-susceptibility (or the respective refractive indices, Δn) that subsequently results in the effective modulation of the phase dispersion of the incident THz beam.

## Discussions

When operated at resonance condition, a piezoelectric material encounters two consecutive nodes: a resonant and an antiresonant frequency node. At the resonant frequency (*f*_*r*_) node, the system sees a minimum series impedence such that, if the resistance caused by mechanical losses is ignored, the impedance of the equivalent electrical circuit describing the element is approximated as zero. As the frequency increases, the impedance increases to a maximum value (minimum admittance). The maximum impedance frequency, termed as the anti-resonant frequency (*f*_*a*_) node, approximates a parallel resonance frequency. At the frequency *f*_*a*_, the parallel impedance in the equivalent electrical circuit is considered to be infinitely large if the contribution to the resistance associated with mechanical losses is ignored. For an optical material, the impedance corresponds to the effective index of refraction. More details on the equivalent electrical circuit of a piezoelectric resonator are provided in Supplemental information S2.

On the other hand, if it is operated near the resonance condition, the phonon vibrations developed from the piezoelectric effect get translated into surface waves which induce changes in the relative refractive index of the material. This change in refractive index modulates the phase of the reflected wave due to the varying optical path encountered by the incident THz wave. An analytical interpretation of this phenomenon can be described as follows:

The normally incident THz wave 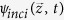, having an angular frequency *ω*_*THz*_, propagating along the z direction with wave vector 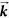 can be represented as


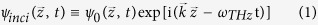


The reflected wave 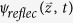, will have an additional phase shift Δφ introduced by the interaction with the modulated surface of the material,


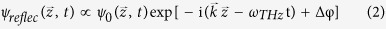


This phase shift can be attributed to the path length difference (ΔL) experienced by the reflected beam subjected to the relative change in refractive index due to the induced surface strain in the material,


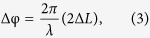


where *λ* is the wavelength of the incident wave. Since both piezoelectric coefficient and electro-optic coefficients are third rank tensor properties, the piezoelectric effect (for a birefringent material LiNbO_3_) will also involve field-induced changes in the optical indicatrix. Thus the path length difference (ΔL) used hereafter takes into consideration the field induced changes in the refractive index, i.e. ΔL(Δn), of the material as well.

From converse piezoelectric effect, the charge coupled strain *x*_*jk*_ can be related to the excitation field *E*_*i*_, where *d*_*ijk*_ is the piezoelectric coefficient of the material,


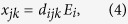


where for longitudinal displacement, the strain *x*_*jk*_ = 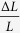 therefore ΔL = L *d*_*ijk*_ *E*_*i*_.

Since the applied a.c. field with excitation frequency 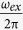 is sinusoidal,


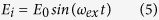


then equation (4) becomes:


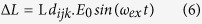


Using (3)


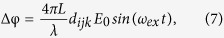


and the reflected wave (Equation 2) becomes


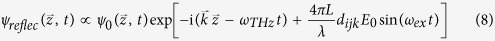


Therefore, the added phase shift introduced in the reflected THz beam is a function of the piezoelectric coefficient (*d*_*ijk*_) of the material involved, which can be tuned by altering the frequency of the external bias a.c. field (*ω*_*ex*_). It is the varying piezoelectric strain leading to induced relative change in refractive index at different excitation frequencies which altered the phase transfer function of the reflected THz beam 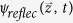. This tuning effect is explicitly observed in Fig. 2(a), where the varying relative phase response is obtained for different frequencies of the applied field (other than 20.8 kHz). The rippling surface acoustic wave that gave rise to this phase shifts is well depicted in Fig. 2(b).

**Figure 4:**
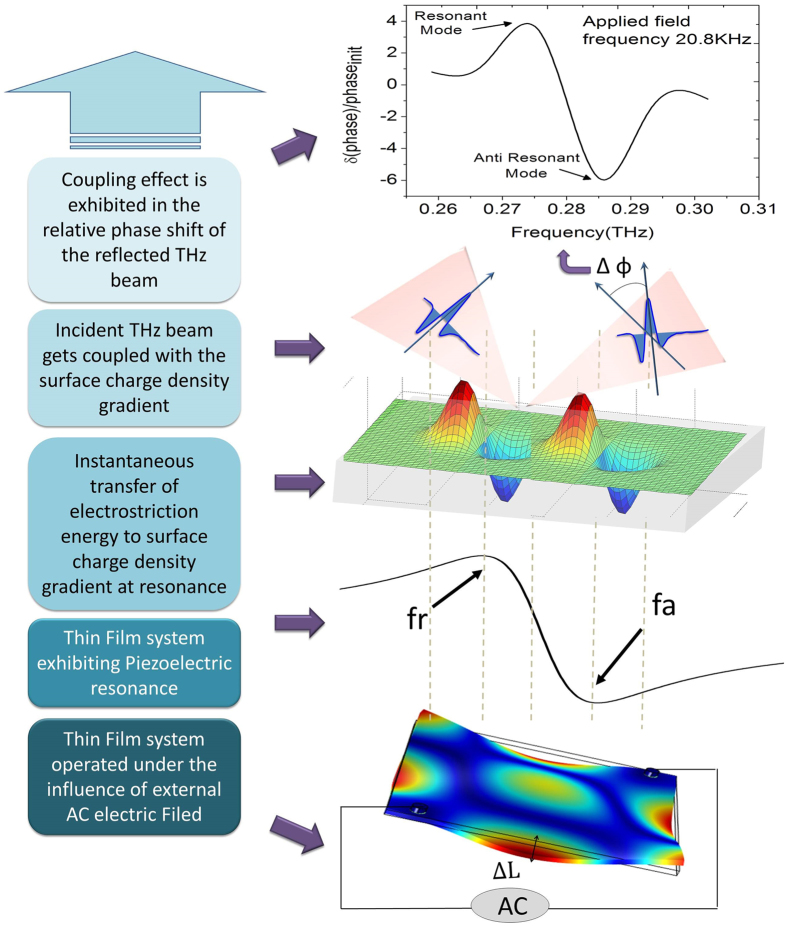
Resonance-defined electric field controlled phase modulation of the incident THz beam. Schematic illustrations explaining the interplay between electromagnetic (EM) and lattice-phonon vibrations at piezo-resonance in a ferroelectric material. When the thin film is operated at the resonance condition (*f*_*r*_ and *f*_*a*_ represents the resonant and the anti-resonant frequency nodes respectively) by applying a low frequency external electric field (with 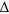
L depicting the path-length difference, experienced by the reflected beam due to the applied electric field), the material density gradient gets instantaneously translated to the surface charge density gradient. The incident THz beam gets coupled with this surface charge density gradient to modulate its phase transfer function, which is prominently depicted in the relative phase change observed in the reflected beam. The resonance and anti-resonance nodes are distinctively captured in the relative phase shift 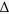
φ when excited at the resonance condition i.e. at 20.8 kHz.

When the applied frequency coincides with a piezoelectric resonance condition (like at 20.8 kHz in this case), the lattice vibrations are accompanied by a varying density of surface charges. For a finite medium having uniform bulk polarization (such as LN in this case where 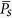//z-axis), although the volumetric charges S_v_ remain constant, the induced stress 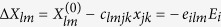 (where *c*_*lmjk*_ and *e*_*ilm*_ are the stiffness and the piezoelectric stress coefficients of the material respectively and, 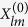 is the initial stress) will account for changes in the bond surface charge density *S_b_* 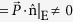 (where 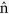//c-axis) as shown in Fig. 4. Considering an applied in-plane field, the lattice vibrations of the *d*_*133*_ mode will be out of plane where the electromechanical factor of the longitudinal waves traveling along the z-axis [001] can be calculated from the modified Christoffel Equation 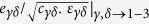 (see further detail in Supplemental information S3). Therefore, the fractional changes in the acoustic phase velocity Δv/v correspond to the fractional changes in material density (ρ) or (Δv)^2^ = *c*/Δρ.

At resonance, the piezoelectric mediated material density change (Δρ) instantaneously gets transferred to a change in surface charge density (without any energy loss), which otherwise will either remain spatially uniform or temporally unable to follow the modulation field, if away from the resonance condition. The depletion or accumulation of the bound surface charges develops a localized electric field E_loc_ 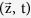 which couples with the polarization of the incident THz wave 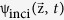 to alter its phase response. The superposition of the electric polarization of the incident THz wave with the polarization direction of E_loc_ 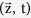 that rotates between out-of to in-to the plane of the piezoelectric resonator, is imprinted into the THz wave with the resultant phase shift of the coupled THz wave. Therefore, the phase modulation of the reflected wave when operating the thin film at resonance can be described as a combination of path-length modulated and polarization mediated modulation defined at the piezoelectric resonance:


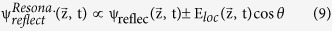


where θ is the angle between the polarization directions of the two electric field components.

The complex coupling effect is illustrated in the relative phase response 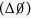 plot of the reflected THz beam when captured at the piezoresonance condition (with the applied excitation field at 20.8 kHz), where the resonant and the anti-resonant nodes are distinctly visible in the captured phase response (as shown in Fig. 4).

Thus here we have demonstrated the evolution of the phase modulation where, when the material was excited far away from the resonance e.g. far below 20.6 kHz, the accumulated effect was not that prominently observable. At near resonance condition (20.6–20.8 kHz & 20.8–20.9 kHz), surface-displacement leads to differential contrast in the relative refractive indices that subsequently results in the effective modulation of the phase dispersion of the incident THz beam. At resonance (20.8 kHz), the combined charge-coupled surface-strain results in the phase reversal.

LiNbO_3_ being a ferroelectric material, it is the inherent hysteretic and highly nonlinear polarization behavior that led to this effectively observable phase-shift in the reflected THz beam, which otherwise will not be that significant if compared with a non-ferroelectric.

We believe this qualitative demonstration of a ‘transducer effect’ where a low frequency (10 s of kHz) E-field was used to produce an electromechanical coupling that governs the interactions of waves, lattice vibrations and electromagnetic (EM) terahertz waves, can potentially set the foundation for highly efficient millimeter wave phase controlled devices.

While the piezoelectric-resonance controlled THz phase modulation is reported in this work, we expect that a proper design of the dimension and a tailored choice of ferroelectric materials will extend its implementation to a wide range of phase and amplitude controlled THz devices, including precision controlled broadband and narrowband THz phase modulators, real-time THz switches, and THz imaging projectors with an array of crystalline modules operating at tunable piezo-resonance, as a few examples.

In conclusion, we have experimentally demonstrated a matter-wave interrelations defined by the piezoelectric resonance of a crystalline material (ion-sliced z-cut LiNbO_3_) that leads to the phase modulation of an incident EM wave in the frequency range of 200 GHz–3 THz. The THz beam modulation is achieved by operating the crystalline ferroelectric material at piezo-resonance by exciting it with a low frequency (10 s of kHz) electric field (the frequencies used, lie in between 19.2–20.9 kHz). The terahertz response is expressed in the form of phase-modulation of the reflected THz wave, which shows prominent correspondence for excitation field frequencies near the resonance (20.6 kHz–20.9 kHz with a phase reversal at 20.8 kHz, the resonance condition in this configuration). The terahertz spectral window that reciprocated to the external excitation was found to lie in the range of 0.1–0.5 THz. The relative phase response increases in amplitude from ~0.259 to 0.276 THz, with a phase reversal as well as a maximum response recorded at 0.285 THz. The response mostly vanishes beyond 0.302 THz for the given piezoelectric resonance mode excited. The maximum out-of-plane surface displacement was found, as measured by vibrometer, to be near 20.7 kHz indicating it to be the regime of one of the fundamental modes of the piezoelectric-resonance frequency. This result was further substantiated by the FEA simulations. The simulated results showed that the primary mode of resonance is at 21.2 kHz (which deviated from the experimental observation only by ~2%). It has been further revealed that when at resonance, it is the combined effect of both the change in path-length, introduced by the applied electric field, and the polarization mediated components, attributed by the surface charge density gradient, that shape the phase transfer function of the reflected THz beam. The phase modulator architecture thus demonstrated holds scalability over the amount of phase-change required and the THz frequency window intended for modulation, which can be dynamically tuned based on the frequency of the excitation field. The design can facilitate the fabrication of the envisioned resonance-defined highly sensitive and extremely low energy sub-millimeter wave sensors and modulators. Moreover, its room temperature platform and its relative simplicity of the fabrication process project its suitability for broader range of applications.

## Methods

### Sample configuration and Terahertz characterization

The multilayered thin film system (as acquired from Nanoln Electronics (Jinan, China)) studied in this work is comprised of an ion-sliced single crystal z-cut LiNbO_3_ (LN) of 504 nm deposited on platinized silica grown on an LN substrate of 500 um. A THz time domain spectrometer system, the Mini-Z THz-TDS Spectrometer by Z-Omega, has been employed for the terahertz characterization. As detailed in one of our previous works^36^, it uses an external fiber coupled 1.5 μm femtosecond pulsed laser with duration <100 fs as the optical source. The laser pulses having an average power >100 mW and a repetition rate of 100 MHz illuminate a photoconductive dipole antenna fabricated on a low temperature-GaAs wafer, to generate THz pulses. It has a bandwidth of 3 THz with the peak THz frequency lying ~0.4 THz. An ITO coated glass at the detector module combines the the laser pulses with the imprinted THz signal. The spatial and temporal overlap of the two signals, as captured by the Pockels electro-optic effect of a GaAs crystal, is recorded by a pair of InGaAs balanced photodiodes as the resultant temporal THz response. Standard reflection configuration of the system at near normal incidence was used to acquire the reflection spectra, with a polished silver mirror used for capturing the reference reflection beam.

Based on a previous study^36^ where an elaborate set of THz analysis have been performed to extract the material properties of different crystalline orientations of electroded and non electroded LN thin film systems, the configuration used in this project was selected as it showed the most promise for fullfilling the requirements of the current device design.

### Vibrometer experimental details

The piezoresonance frequency measurements were conducted using an UHF-120 Polytec Vibrometer (Irvine, CA). It works on Laser Doppler Effect and is based on an interferometry technique, using which non-contact vibration measurements were performed on the sample surface. An a.c. E-field of 10 V p-p at various frequencies was applied across the surface of the sample via two silver electrodes situated at two opposite corners using an Agilent 33220a waveform generator. The laser beam from the Laser Doppler Vibrometer (LDV) was directed to the surface of the sample and the vibrational amplitude and frequency response were obtained from the Doppler shift of the reflected laser beam with respect to the internal reference. The LDV counts the bright-dark fringes on the detector and directly measures the displacement as well as the vibrational velocity. The frequency shift measured was introduced due to the displacement of the sample surface. Using suitable interpolation techniques along with digital demodulation, a resolution down to the pm range was achieved. The Polytech Vibrometer was conjugated with a WavePro 725Zi LeCroy Oscilloscope (Chestnut Ridge, NY) and a SMBV100A Rohde & Schwarz–Vector Signal Generator (Munchen, Germany) to cover the intended range of frequencies.

### AFM and XRD measurements

AFM topographical scans (256 × 256 pixels) were performed using Multimode V Scanning Probe Microscope configured in tapping mode and equipped with standard rectangular shaped SCM-PIT probes (supplied by Bruker). The probes have a radius of ~20 nm and a spring constant of 2.8, mounted on a 2.75 μm thick rectangular shaped antimony doped Silicon cantilever.

The X-ray θ–2θ diffraction spectrum was acquired using Shimadzu 6000 X-ray Diffractometer. The Cu K-alpha radiation line was used for the purpose, operated in a step scan mode of 0.02 per step. CIF file no 1521772 (available in Crystallography Open Database) was referred to for the indexing of the angular spectrum.

### Finite element simulation

Finite element simulations were performed using the commercially available package of COMSOL Multiphysics 4.4. To simulate the structure, the geometry of the top-electrode (diagonally placed at the two corners of the film surface) multi-layered thin-film was recreated with domains defined accordingly. The piezoelectric devices module was used with the constitutive relation of the piezoelectric material properties set at stress-charge form. The fixed constraints were applied to the boundaries comprising the electrodes, with one of the electrodes defined as the terminal and the other as the ground. A frequency domain study was performed with the driving frequency sweeping in the range of 19 kHz to 25 kHz using the direct stationary solver PARDISO. In all the simulations pre-defined fine meshing was employed.

## Additional Information

**How to cite this article**: Dutta, M. *et al*. Low frequency piezoresonance defined dynamic control of terahertz wave propagation. *Sci. Rep.* **6**, 38041; doi: 10.1038/srep38041 (2016).

**Publisher's note:** Springer Nature remains neutral with regard to jurisdictional claims in published maps and institutional affiliations.

## Supplementary Material

Supplementary Information

## References

[b1] Carr G. L. . High-power terahertz radiation from relativistic electrons. Nature 420, 153–156 (2002). 12432385 10.1038/nature01175

[b2] Byrd J. M. . Observation of broadband self-amplified spontaneous coherent terahertz synchrotron radiation in a storage ring. Physical Review Letters 89 (2002). 10.1103/PhysRevLett.89.224801 12485072

[b3] Abo-Bakr M., Feikes J., Holldack K., Wustefeld G. & Hubers H. W. Steady-state far-infrared coherent synchrotron radiation detected at BESSY II. Physical Review Letters 88 (2002). 10.1103/PhysRevLett.88.254801 12097089

[b4] Doria A., Gallerano G. P., Giovenale E., Messina G. & Spassovsky I. Enhanced coherent emission of terahertz radiation by energy-phase correlation in a bunched electron beam. Physical Review Letters 93 (2004). 10.1103/PhysRevLett.93.264801 15697983

[b5] Leemans W. P. . Observation of terahertz emission from a laser-plasma accelerated electron bunch crossing a plasma-vacuum boundary. Physical Review Letters 91 (2003). 10.1103/PhysRevLett.91.074802 12935022

[b6] Shibata Y. . Observation of Coherent Transition Radiation at Millimeter and Submillimeter Wavelengths. Physical Review A 45, R8340–R8343 (1992). 10.1103/physreva.45.r8340 9907017

[b7] Korbly S. E., Kesar A. S., Sirigiri J. R. & Temkin R. J. Observation of frequency-locked coherent terahertz Smith-Purcell radiation. Physical Review Letters 94 (2005). 10.1103/PhysRevLett.94.054803 15783652

[b8] Takahashi T. . Observation of coherent Cerenkov radiation from a solid dielectric with short bunches of electrons. Physical Review E 62, 8606–8611 (2000). 10.1103/physreve.62.8606 11138160

[b9] Dai J., Xie X. & Zhang X. C. Detection of broadband terahertz waves with a laser-induced plasma in gases. Physical Review Letters 97 (2006). 10.1103/PhysRevLett.97.103903 17025819

[b10] Shi W., Ding Y. J. J., Fernelius N. & Vodopyanov K. Efficient, tunable, and coherent 0.18–5.27-THz source based on GaSe crystal. Optics Letters 27, 1454–1456 (2002). 18026477 10.1364/ol.27.001454

[b11] Stepanov A. G., Bonacina L., Chekalin S. V. & Wolf J. P. Generation of 30 mu J single-cycle terahertz pulses at 100 Hz repetition rate by optical rectification. Optics Letters 33, 2497–2499 (2008). 18978899 10.1364/ol.33.002497

[b12] Hirori H., Doi A., Blanchard F. & Tanaka K. Single-cycle terahertz pulses with amplitudes exceeding 1 MV/cm generated by optical rectification in LiNbO_3_. Applied Physics Letters 98 (2011).

[b13] Ruchert C., Vicario C. & Hauri C. P. Scaling submillimeter single-cycle transients toward megavolts per centimeter field strength via optical rectification in the organic crystal OH1. Optics Letters 37, 899–901 (2012). 22378431 10.1364/OL.37.000899

[b14] Xu J. Z., Zhang C. L. & Zhang X. C. Recent progress in terahertz science and technology. Progress in Natural Science 12, 729–736 (2002).

[b15] Ferguson B. & Zhang X. C. Materials for terahertz science and technology. Nature Materials 1, 26–33 (2002). 12618844 10.1038/nmat708

[b16] Federici J. & Moeller L. Review of terahertz and subterahertz wireless communications. Journal of Applied Physics 107 (2010).

[b17] Chan W. L., Deibel J. & Mittleman D. M. Imaging with terahertz radiation. Reports on Progress in Physics 70, 1325–1379 (2007).

[b18] Leahy-Hoppa M. R., Fitch M. J., Zheng X., Hayden L. M. & Osiander R. Wideband terahertz spectroscopy of explosives. Chemical Physics Letters 434, 227–230 (2007).

[b19] Kleine-Ostmann T. & Nagatsuma T. A Review on Terahertz Communications Research. Journal of Infrared Millimeter and Terahertz Waves 32, 143–171 (2011).

[b20] Kampfrath T. . Coherent terahertz control of antiferromagnetic spin waves. Nature Photonics 5, 31–34 (2011).

[b21] Qi T. T., Shin Y. H., Yeh K. L., Nelson K. A. & Rappe A. M. Collective Coherent Control: Synchronization of Polarization in Ferroelectric PbTiO3 by Shaped THz Fields. Physical Review Letters 102 (2009). 10.1103/PhysRevLett.102.247603 19659049

[b22] Rahm M., Li J. S. & Padilla W. J. THz Wave Modulators: A Brief Review on Different Modulation Techniques. Journal of Infrared Millimeter and Terahertz Waves 34, 1–27 (2013).

[b23] Padilla W. J., Taylor A. J., Highstrete C., Lee M. & Averitt R. D. Dynamical electric and magnetic metamaterial response at terahertz frequencies. Physical Review Letters 96 (2006). 10.1103/PhysRevLett.96.107401 16605787

[b24] Kleine-Ostmann T., Dawson P., Pierz K., Hein G. & Koch M. Room-temperature operation of an electrically driven terahertz modulator. Applied Physics Letters 84, 3555–3557 (2004).

[b25] Driscoll T. . Dynamic tuning of an infrared hybrid-metamaterial resonance using vanadium dioxide. Applied Physics Letters 93 (2008).

[b26] Sensale-Rodriguez B. . Broadband graphene terahertz modulators enabled by intraband transitions. Nature Communications 3 (2012). 10.1038/ncomms1787 22510685

[b27] Fekete L., Kadlec F., Kuzel P. & Nemec H. Ultrafast opto-terahertz photonic crystal modulator. Optics Letters 32, 680–682 (2007). 17308600 10.1364/ol.32.000680

[b28] Kersting R., Strasser G. & Unterrainer K. Terahertz phase modulator. Electronics Letters 36, 1156–1158 (2000).

[b29] Hsieh C. F., Pan R. P., Tang T. T., Chen H. L. & Pan C. L. Voltage-controlled liquid-crystal terahertz phase shifter and quarter-wave plate. Optics Letters 31, 1112–1114 (2006). 16625920 10.1364/ol.31.001112

[b30] Chen H. T. . A metamaterial solid-state terahertz phase modulator. Nature Photonics 3, 148–151 (2009).

[b31] White R. . Tunable single frequency ultraviolet generation from a continuous wave Ti:sapphire laser with an intracavity PPLN frequency doubler. Appl. Phys. B: Lasers and Optics 77, 57–550 (2003).

[b32] Huang C. Y., Bhalla A. S. & Guo R. Y. Measurement of microwave electro-optic coefficient in Sr_0.61_Ba_0.39_Nb_2_O_6_ crystal fiber. Applied Physics Letters 86 (2005).

[b33] Huang C. Y., Guo R. Y. & Bhalla A. S. Real-time observation of pulse reshaping using Sr_0.61_Ba_0.39_Nb_2_O_6_ single crystal fiber in a microwave cavity. Applied Physics Letters 86 (2005).

[b34] McIntosh R., Garcia C., Bhalla A. S. & Guo R. Y. Periodically Poled Structure on Microwave Transmissions Evaluated by Scattering Parameters Integrated Ferroelectrics 131, 219–229 (2011).

[b35] Jiang J., Meng X. J., Geng D. Q. & Jiang A. Q. Accelerated domain switching speed in single-crystal LiNbO_3_ thin films. Journal of Applied Physics 117 (2015).

[b36] Dutta M., Ellis C., Peralta X. G., Bhalla A. & Guo R. Y. Terahertz electrical and optical properties of LiNbO_3_ single crystal thin films. Proc Spie 9586 (2015).

