# Supplementary material for: Low frequency piezoresonance defined dynamic control of terahertz wave propagation
Source: Sci Rep. 2016 Nov 30;6:38041. doi: 10.1038/srep38041 (PMC5128798; doi:10.1038/srep38041)
Supplement: Supplementary Information [file srep38041-s1.pdf]

Low frequency piezo-resonance defined dynamic control of terahertz wave propagation

Moumita Dutta[1]*, Soutik Betal[1], Xomalin G. Peralta[2], Amar S. Bhalla[1] and Ruyan Guo[1]

[1] *Department of Electrical and Computer Engineering, University of Texas at San Antonio, San Antonio, TX 78249, USA*
[2] *Department of Physics & Astronomy, University of Texas at San Antonio, San Antonio, TX 78249, USA*

Supplemental information S1.Initial THz characterizations of different Lithium Niobate thin film systems to analyse their suitability for the device design

Z-cut and x-cut ion-sliced Lithium Niobate thin films with and without embedded electrodes have been investigated employing terahertz spectroscopy both in transmission and reflection mode along with z-cut single crystal in bulk form to identify the potential material configuration as per the requirement of the application targeted[1].

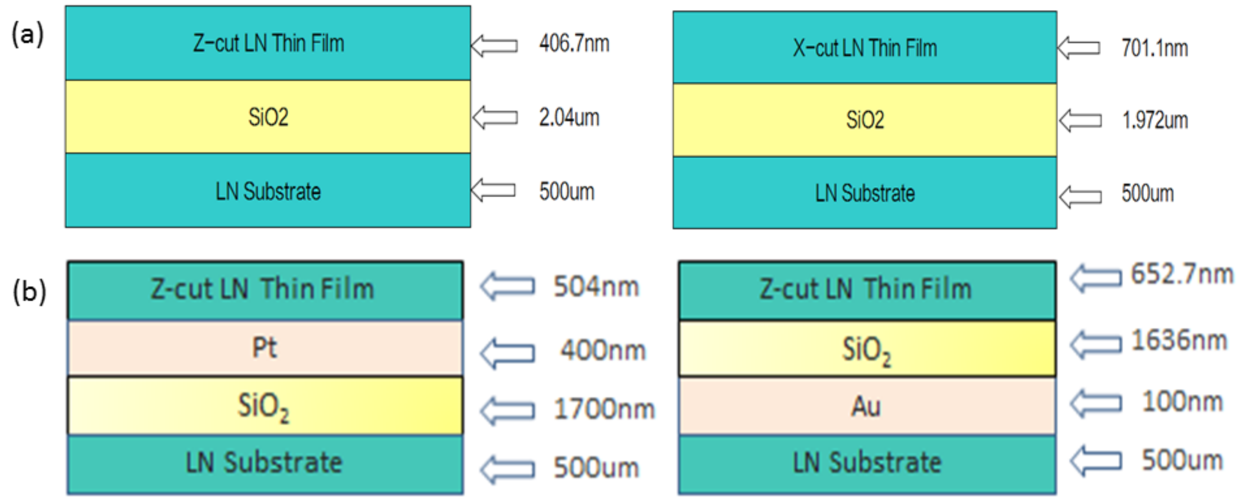

Supplemental Figure1: LN thin Films studied-(a) non-electroded z-cut (left) and x-cut (right) (b) electroded with platinum (left) and gold (right)

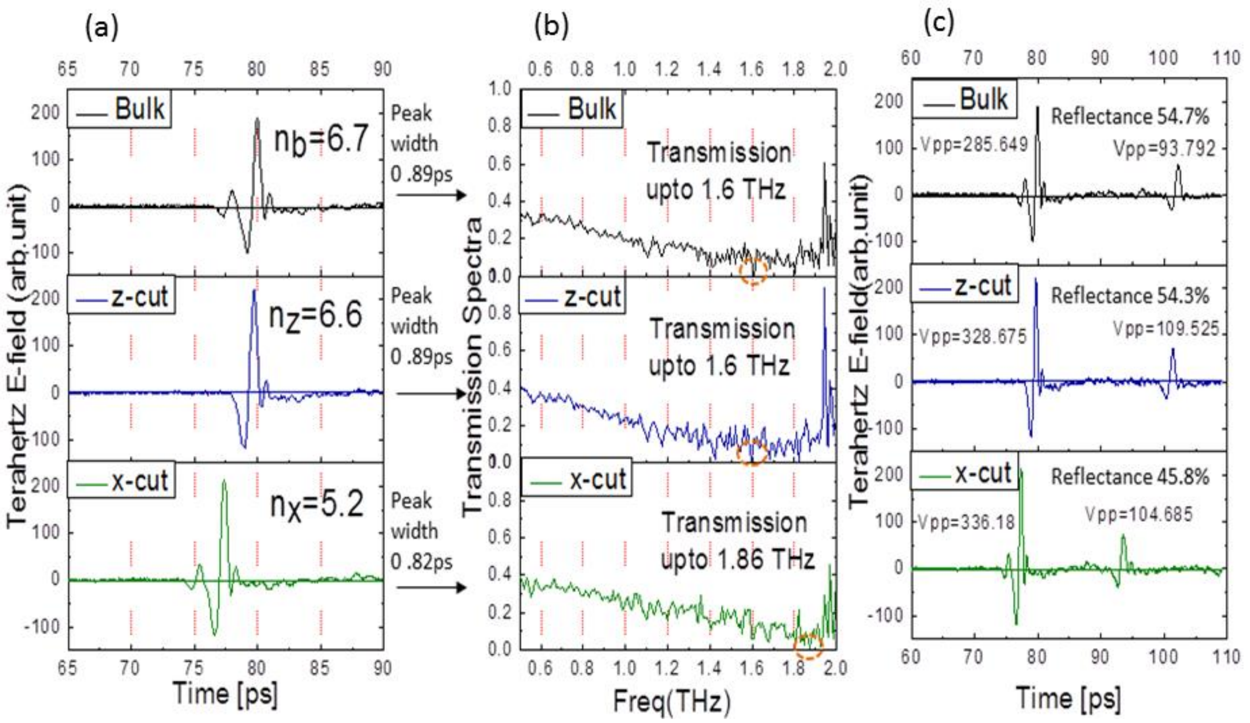

Supplemental Figure2: Correspondence of (a) refractive index (n) with the time delay of the time domain waveforms, (b) Peak widths with the transmission bandwidth in frequency domain, (c) Peak-to peak E-field (Vpp) of the post pulse with the reflectance; for bulk, non-electroded z-cut and x-cut LN.

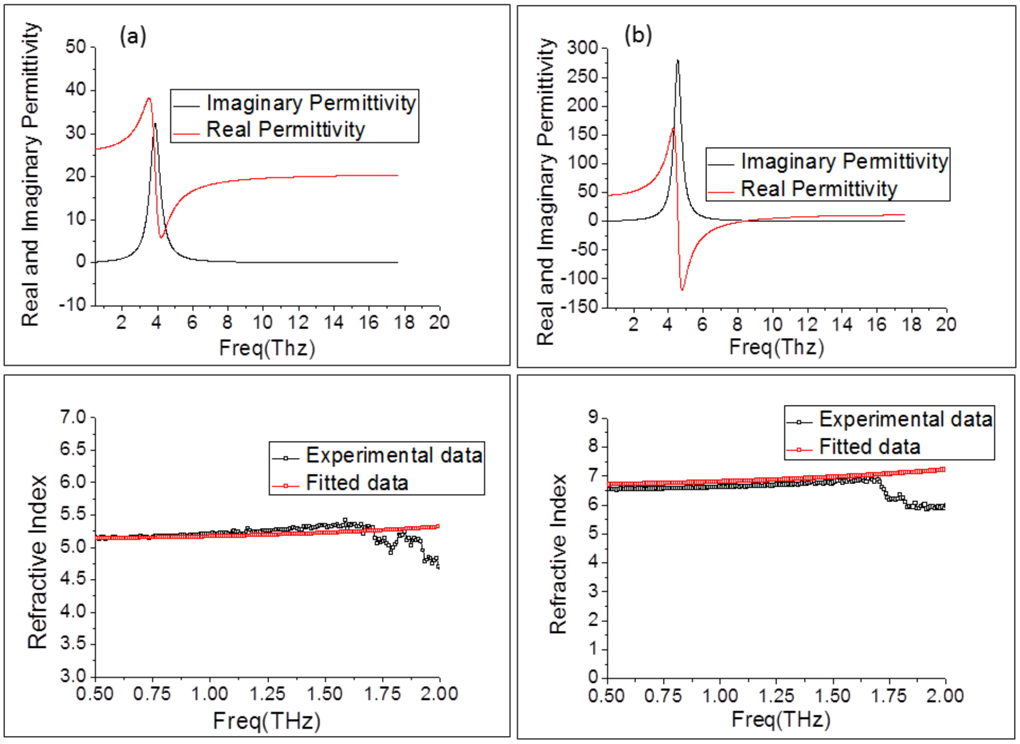

Supplemental Figure3: Complex Dielectric Constants obtained from the oscillator model (top) and the experimental data along with the fitted refractive index (bottom) for non-electroded (a) x-cut and (b) z-cut.

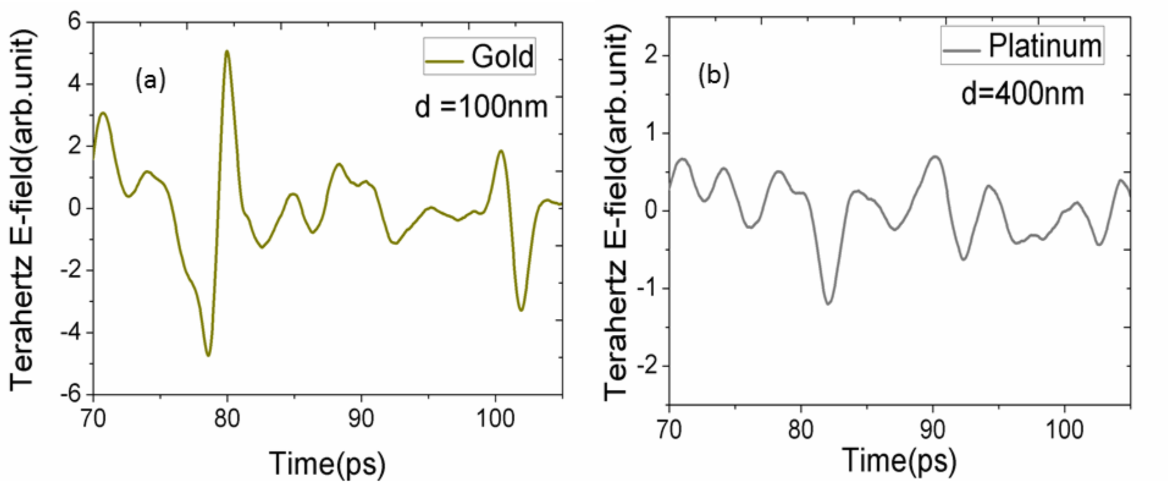

Supplemental Figure4: Transmission spectra of electroded LN thin films with embedded (a) Gold and (b) Platinum (with d as the respective thickness of the electrode).

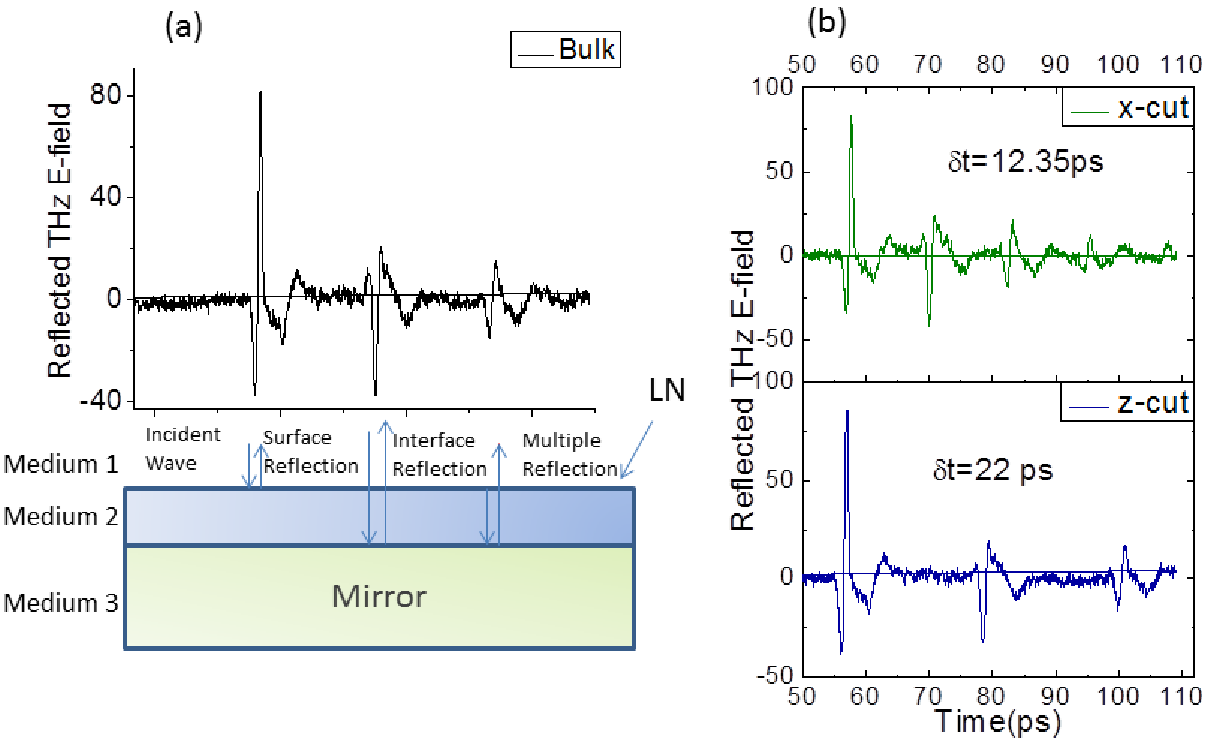

Supplemental Figure5: Reflection spectra of (a) bulk and (b) x-cut & z-cut LN thin films(where δt is the delay between the primary pulse and the first reflection) .

Supplemental information S2. Equivalent electrical circuit for piezoelectric resonators

A Piezoelectric resonator can be described by the simplified equivalent circuit as shown in Supplemental Figure6,where the resultant admittace can be defined as Y= $j\omega C_0+\frac{1}{(R_1+j\omega L_1+\frac{1}{j\omega C_1})}$ and the mechanical Quality

Qm=$\frac{f_n{}^2}{2\pi f_m C_0 Z_m(f_n{}^2 - f_m{}^2)}$,where $f_n$and $f_m$are the minimum and maximum impedence frequencies and Z_m is the maximum impedence.

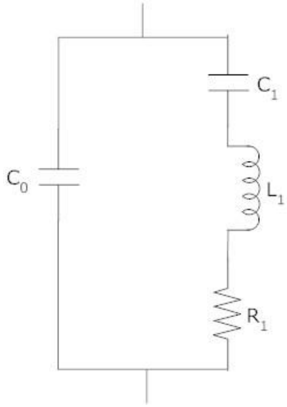

Supplemental Figure6: Simplified equivalent electrical circuit of a Piezoelectric resonator

Supplemental information S3. Modified Christoffel Equation

Wave propagation in piezoelectric media involves a coupled solution of the mechanical equations of motion and Maxwell's equations for electromagnetic waves. If Newton's second law and Maxwell's equation for the electromagnetic waves with no free charges present are considered, then for plane waves the elastic wave velocities u, can be solved from the following equation (where ρ , c and e are the density, elastic coefficient and piezoelectric stress coefficient of the material with the wave vector components k= (ω/v) N (where v, ω and N are the velocity, angular frequency and direction cosines of the wave normal[2].

$$\left[\frac{1}{\rho} c_{ijkl} N_j N_l + \frac{1}{\rho} \frac{(N_m e_{mij} N_j)}{N_m \varepsilon_{mj} N_j} - v^2 \delta_{ik}\right] u_k = 0$$

The first term corresponds to the Christoffel Equation, and the second term to the piezoelectric interaction.

1. Dutta, M., et al., *Terahertz electrical and optical properties of LiNbO3 single crystal thin films.* Photonic Fiber and Crystal Devices: Advances in Materials and Innovations in Device Applications Ix, 2015. **9586**.
2. E.Newnham, R., *Properties of materials:Anisotropy, Symmetry, Structure.* Oxford,University Press, 2005.
